# Four years into the Indian ocean field epidemiology training programme

**DOI:** 10.11604/pamj.2017.26.195.10358

**Published:** 2017-04-04

**Authors:** Ariane Halm, Thomas Seyler, Sainda Mohamed, Saindou Ben Ali Mbaé, Armand Eugène Randrianarivo-Solofoniaina, Maherisoa Ratsitorahina, Ram Nundlall, Shahina Aboobakar, Jastin Bibi, Laurent Filleul, Patrice Piola, Harimahefa Razafimandimby, Harena Rasamoelina, Marta Valenciano, Alain Moren, Eric Cardinale, Richard Lepec, Loïc Flachet

**Affiliations:** 1 Health Surveillance Unit, SEGA One Health Network, Indian Ocean Commission, Mauritius; 2 Epidemiology Department, EpiConcept, France; 3 Surveillance Unit, World Health Organisation, Union of the Comoros; 4 National Epidemiological Surveillance Unit, Ministry of Health, Union of the Comoros; 5 Epidemiological Surveillance Department, Ministry of Health, Madagascar; 6 Epidemiology Unit, Pasteur Institute, Madagascar; 7 Disease Surveillance and Response Unit, Ministry of Health, Seychelles; 8 French Regional Epidemiology Unit (CIRE), Reunion, France; 9 Communicable Diseases Control Unit, Ministry of Health and Quality of Life, Mauritius; 10 French Agricultural Research Centre for International Development (CIRAD), Exotic and Emerging Animal Disease Control Research Unit (CMAEE UMR), Cyroi platform, Reunion, France

**Keywords:** FETP, regional surveillance, epidemiology training, Public Health preparedness

## Abstract

**Introduction:**

Following the 2005-6 chikungunya outbreak, a project to strengthen regional Public Health preparedness in the Indian Ocean was implemented. It includes the Comoros, Madagascar, Mauritius, Reunion (France) and Seychelles. A Field Epidemiology Training Programme (FETP-OI) was started in 2011 to develop a pool of well-trained intervention epidemiologists.

**Methods:**

The FETP-OI consists of two years of supervised, learning-by-doing, on-the-job training at national sites involved in disease surveillance and response. It includes work placements at the Madagascar Pasteur Institute and the French regional epidemiology unit in Reunion and up to three training courses per year. Training objectives include epidemiological surveillance, outbreak investigations, research studies, scientific communication and transfer of competencies.

**Results:**

In four years, two cohorts of in total 15 fellows originating from four countries followed the FETP-OI. They led 42 surveillance projects (71% routine management, 14% evaluations, 12% setup, 3% other) and investigated 36 outbreak alerts, 58% of them in Madagascar; most investigations (72%) concerned foodborne pathogens, plague or malaria. Fellows performed 18 studies (44% descriptive analyses, 22% disease risk factors, and 34% on other subjects), and presented results during regional and international conferences through 26 oral and 15 poster presentations. Four articles were published in regional Public Health bulletins and several scientific manuscripts are in process.

**Conclusion:**

The FETP-OI has created a regional force of intervention consisting of field epidemiologists and trained supervisors using the same technical language and epidemiological methods. The third cohort is now ongoing. Technically and financially sustainable FETP-OI projects help addressing public health priorities of the Indian Ocean.

## Introduction

**FETPs in the world**: The United States Centers for Disease Control and Prevention (US CDC) created the first Field Epidemiology Training Programme (FETP) in 1951. It was called Epidemic Intelligence Service (EIS) and its core is a “learning by doing” approach [1, 2]. Following its success, FETPs modelled from the US EIS were created in numerous countries over the following decades. In the 1990s, the European Programme for Intervention Epidemiology Training (EPIET) was created [3]. It was the first multinational and multicultural FETP, where fellows moved to another country for the duration of the training programme. In its beginning in 1995, EPIET comprised 13 countries [4], today, numerous host institutes in 28 countries form part of the network (http://ecdc.europa.eu/en/epiet/institutes/Pages/institutes.aspx). The Training Programs in Epidemiology and Public Health Interventions Network (TEPHINET) was founded in 1997 to improve networking between programmes [5]. Today, the TEPHINET network comprises 63 FETPs in 88 countries around the world (http://www.tephinet.org/). In Africa several national or regional FETPs were set up between 1990 and 2010 (Zimbabwe, Uganda, Kenya, South Africa, Tanzania, Nigeria, Ethiopia, West Africa, etc.), and they have generally shown a good retention of graduates in their home countries [6]. To our knowledge, most FETP trainings have a duration of two years and resemble each other in content and objectives, dedicated to training individuals who are likely to make field epidemiology their career and harmonising expertise and technical language beyond borders. Their main goal is to train competent field epidemiologists to strengthen the Public Health workforce as well as systems for disease surveillance, alert and response. An important additional asset of these programmes is the creation of solid collaboration and exchange networks [7-9].

The output, use and recognition of FETPs, as well as their importance in harmonising methods, establishing evidence and gathering Public Health information has been acknowledged [8, 10, 11]. With the start of the One Health initiative, Public Health programmes including FETPs have started integrating environmental, human and veterinary health issues [12].

**Figure 1:**
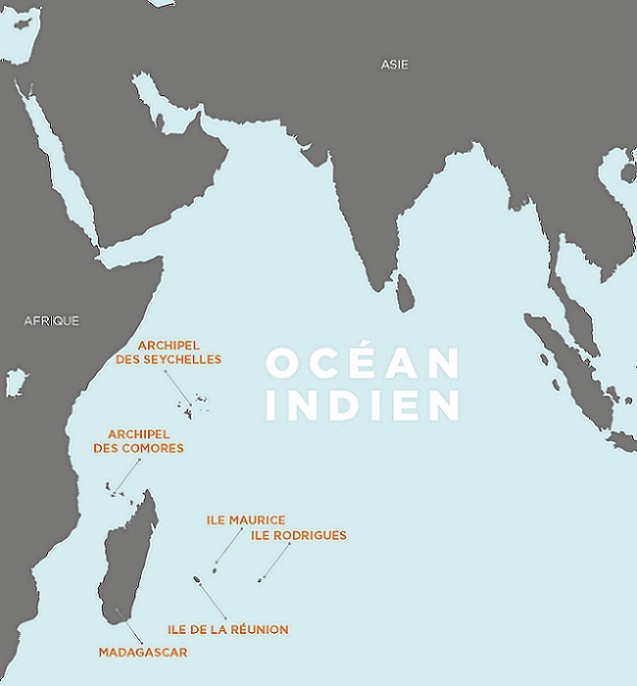
Map of the Indian Ocean and countries part of the IOC, IOC annual report 2014 (French)

**Indian Ocean & IOC:** Five countries in the Indian Ocean have established formal and informal exchanges on different fields (diplomacy, health, economical infrastructures, regional growth, climate change, human and natural resources, etc) which are moderated by the Indian Ocean Commission (IOC, www.commissionoceanindien.org/, Figure 1. They include the Comoros, Madagascar, Mauritius, Reunion (France) and Seychelles, a highly diverse group of nations in terms of economy, size, population, culture, and religion. There are equally important differences in Public Health resources and practice between them. The Indian Ocean countries are prone to outbreaks at national and regional level due to internal traffic and exchanges as well as those with mainland Africa, Asia and the rest of the world [13]. In 2005-6, the region experienced a large chikungunya outbreak [14], and there have been repeated outbreaks of dengue-like syndrome as well as the influenza pandemic 2009. While the Comoros have undergone effective malaria elimination efforts [15], Madagascar still has a high incidence of malaria [16] and is home to the world's highest number of plague cases [17]. Emerging diseases like Rift Valley fever [18, 19], West Nile virus [20], leptospirosis [21], cysticercosis are being identified and studied in the Indian ocean region.

**Public Health information and alert system & SEGA**: The Public Health Surveillance, Alert and Response network SEGA (Surveillance Epidémiologique et Gestion d'Alertes) One Health was set up in 2009 with the aim of strengthening regional public health preparedness through network creation and capacity reinforcement in applied epidemiology, and of harmonising regional Public Health response. It is coordinated by the Surveillance and Response unit of the IOC. SEGA aims to coordinate surveillance, alert, investigation and response to epidemics in the Indian Ocean and to reinforce capacity in the national health ministries of IOC Member States. The French Development Agency (Agence Française de Développement) funds the project. In 2011, the FETP-OI was set up within this project. Its objectives are to develop a pool of intervention epidemiologists, increase the number and competences of public health professionals and future leaders in epidemiological surveillance and response within the national ministries. The FETP-OI aims to reinforce exchange and communication between the IOC member countries and render the regional network solid and sustainable. The FETP-OI was officially affiliated to TEPHINET in 2015.

## Methods

The FETP-OI consists of two years of supervised, learning-by-doing, on-the-job training at national or regional sites involved in disease surveillance and response in the IOC Member States Table 1. It is a full-time activity, and like for other FETPs, field epidemiology is the core part of the programme, prioritising practical experience over theory. The organisation and content of the programme are modelled on those of the US EIS, and the European EPIET. During their two year training, in addition to the work carried out in their respective host institute, the FETP-OI fellows have to undertake two three-month work placements at the Pasteur Institute in Madagascar (IPM) and the French regional epidemiology unit (CIRE) in Reunion (France). These allow fellows to gain competences in laboratory methods and to actively participate in surveillance, investigation, response and research activities in different settings and epidemiology teams. The FETP-OI starts with a three-week residential intensive introductory course on basic methods in epidemiology. During this period, one week is dedicated to basic epidemiological concepts, one to outbreak investigation, and one to epidemiological surveillance. There are theoretical presentations but the large part of the course consists of case studies and group work with feedback constituting practical exercises in teamwork and communication. Following this, there are four more training modules (analysis of surveillance data, outbreak investigation, project review, scientific communication) that serve as advanced theoretical basis. All fellows present their projects' results during regional or international conferences to ensure scientific communication of methods and results. The programme targets individuals working in Public Health interested in field epidemiology. The IOC Member States submit a shortlist of candidates before a selection committee makes the final decision.

**Table 1:** institutions directly participating in the FETP-OI, 2011-15

Name	Country
Direction Nationale de la Santé (DNS), Ministère de la Santé	Comoros
Directions Régionales de la Santé (DRS) de Ndzouani et Mwali	
Direction de Veille Sanitaire et Surveillance Epidémiologique (DVSSE), Ministère de la Santé	Madagascar
Institut Pasteur de Madagascar (IPM)	
Communicable Diseases Control Unit (CDCU), Ministry of Health	Mauritius
Unité de Veille Sanitaire (UVS), Réseau SEGA One Health, Commission de l’océan Indien	
Cellule interrégionale d'épidémiologie (Cire) de la Réunion	Reunion (France)
Centre de coopération internationale en recherche agronomique pour le développement (CIRAD) de la Réunion	
Disease Surveillance and Response Unit (DSRU), Ministry of Health	Seychelles

During the training the fellows should achieve five pedagogical objectives: (A) work on an epidemiological surveillance project involving either the set-up, management (data collection, analysis and feedback) or evaluation of a surveillance system, (B) carry out an outbreak investigation, (C) perform an epidemiological study, (D) make a scientific communication of their project results, and (E) transfer competencies by teaching epidemiology to other public health professionals. There is no formal degree once fellows have reached their training objectives, but each receives a diploma signed by the Secretary General of the IOC as well as attendance certificates for the training courses. A full-time FETP-OI coordinator is in charge of supervising the fellows' work, supporting the achievement of pedagogical objectives, organising and conducting the training courses, and facilitating exchange. Each host site has a dedicated supervisor who helps the FETP fellow to achieve the pedagogical objectives through identifying and supervising work projects and ensuring suitable work conditions. The trio FETP-OI fellow, host site supervisor and programme coordinator have regular exchanges to keep up to date and ensure progress and success of the individual fellowships. The FETP-OI has a pedagogical steering committee composed of SEGA One Health network's national focal points, host site supervisors, World Health Organization representatives, the FETP-OI coordinator, epidemiologists of the surveillance and response unit as well as of the consortium coordinating the project. Its main responsibilities are to define the general orientation, validate the training content and selection criteria for host sites and FETP-OI candidates, validate and deliver the FETP diplomas and facilitate relationships between the different partners.

## Results

During the first four years (2011-15), two cohorts consisting of 15 fellows from four countries have undergone the 24 months of the FETP-OI training: three from the Comoros, eight from Madagascar, two from Mauritius and two from Seychelles.

**Figure 2:**
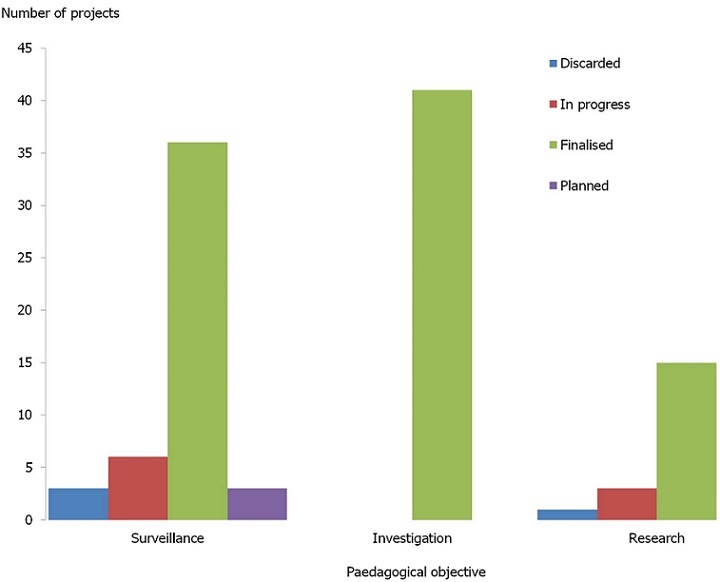
FETP-OI projects fellows have worked on by pedagogical objective (N= 108) and advancement status, Indian Ocean Commission Member States, 2011-5

**Training activities and achievements**: These 15 fellows have in total worked on 108 projects of which 101 (93%) have been finalised or are ongoing. Out of these 37 (37%) have led to scientific communication of project results Figure 2,Table 2.

**Table 2:** FETP-OI projects* (N=101), Indian Ocean Commission member states, 2011-5

		Comoros	Madagascar	Mauritius	Reunion (France)	Seychelles	Total
**Total projects**							
	Surveillance	7	18	5	8	4	42
	Investigation	6	25	3	3	4	41
		2	3	1	9	2	17
**Surveillance**	Research						
	Set-up/ management	1	2		2		5
	Management	5	12	5	4	4	30
	Evaluation	1	3		2		6
			1				1
**Outbreak investigation**	Other						
	Individual	5	21	3	3	4	36‡
**Epidemiological study**	investigations						
	Descriptive analysis			1	7		8
	Risk factors		1	1		2	4
	Other	2	2		2		6
		2	3	1	9	2	18
**Scientific communication**¤	Total						
Oral	International conference	1	3				4
	Regional conference	1	3	1	2	1	8
	Regional scientific seminar	2	8	1	1	2	14
Poster	International conference		6				6
	Regional conference			1	1	1	3
	Regional scientific seminar		3		3		6
Written	Regional Public Health bulletin	1	1		1	1	4

* Only includes projects that have been finalised or which are still ongoing

‡ Each investigation only counted once (more than one fellow involved in several investigations)

¤ Only communications that have already been realised

**Epidemiological surveillance**: The FETP-OI fellows worked on 42 epidemiological surveillance projects, of which 71% constituted management of existing surveillance programmes (including data collection, analyses and feedback). Sometimes more than one fellow was involved in these, for example in Madagascar and Seychelles all fellows were (alternatingly with other Ministry of Health colleagues) responsible for being on call and/or preparing the weekly surveillance report. Fifty percent of the surveillance projects focused on syndromic surveillance systems following trends and identifying unexpected health events related to infectious febrile diseases, 17% on influenza surveillance, and 33% included hospital-based surveillance, leptospirosis, plague and diverse other topics (such as foodborne outbreaks, surveillance during a mass gathering event, etc) Table 3.

**Table 3:** Selected project themes by pedagogical objective (epidemiological surveillance, outbreak investigation, studies), FETP-OI, 2011-5 (a,b,c)

**a. Epidemiological surveillance, some of these constituted more than one (fellow’s) work project**	
1.	Set-up and management of the national sentinel fever surveillance system, Comoros
2.	Evaluation of the foodborne outbreak surveillance system, Madagascar
3.	Set-up and management sentinel hospital surveillance and causes of death, Madagascar
4.	Evaluation of the SMS-reinforced IDSR system in two southern regions, Madagascar
5.	Evaluation of the IPM sentinel influenza surveillance system, Madagascar
6.	Set-up of a hospital-based surveillance, Antananarivo, Madagascar
7.	Management of the national surveillance system for influenza-like illness, diarrhoea, dengue-like syndrome and other important Public Health events, Madagascar
8.	Management of the plague surveillance system, Madagascar
9.	Management of the national surveillance system for influenza-like illness, diarrhoea, dengue-like syndrome and other important Public Health events, Mauritius
10.	Tuberculosis surveillance in a prison, Mauritius
11.	Reinforcement of the leptospirosis surveillance, Mauritius
12.	Evaluation of the emergency room surveillance system, Reunion
13.	Surveillance of acute respiratory infections and gastroenteritis in homes for the elderly, Reunion
14.	Influenza surveillance respiratory pathogens naso-pharyngeal sampling, Reunion
15.	Evaluation of the sentinel General Practitioner surveillance system, Reunion
16.	Set-up and management of a surveillance system during the Indian ocean island games, Reunion
17.	Management of the national surveillance system for influenza-like illness, diarrhoea, dengue-like syndrome and other important Public Health events, Seychelles
18.	Surveillance of communicable diseases, Seychelles
**b. Outbreak investigation, some of these constituted more than one (fellow’s) work project**	
1.	Foodborne outbreak following sea turtle meat consumption 2012, Mwali, Comoros
2.	Dengue-like syndrome outbreak 2015, Ndzouani, Comoros
3.	Foodborne outbreak 2011, Antananarivo, Madagascar
4.	Malaria outbreak 2011-2, East coast, Madagascar
5.	Haemorrhagic fever outbreak 2012, Anjozorobe, Madagascar
6.	Plague outbreaks, Beranimbo, Soanierana Ivongo, Tsiroanomandidy, Ankazobe, Arivonimamo Antanetibe, Arivonimamo Miandrandra, Faratsiho, Mamolifoly, Sandrandahy, 2013 and 2014, Madagascar
7.	Unknown disease outbreaks 2014, Ambilobe and Besalampy, Madagascar
8.	High child mortality due to suspected malaria 2015, Farafangana and Ankililoaka, Madagascar
9.	Autochthonous malaria outbreak 2012, Mauritius
10.	Conjunctivitis outbreak 2015, Mauritius
11.	Gastroenteritis outbreak 2013, St André, Reunion
12.	Foodborne outbreak at a school 2014, Reunion
13.	Foodborne outbreak 2012, St Elizabeth, Seychelles
14.	Caterpillar skin rash epidemic 2015, Seychelles
**c. Epidemiological studies**	
1.	Malaria confirmation for fever cases after elimination project, Mwali, Comores
2.	Healthcare utilisation after animal bites, Madagascar
3.	Risk factors for plague, Madagascar
4.	Extended programme for immunisation vaccination coverage evaluation, Madagascar
5.	Detected leptospirosis cases 2005-2014, Mauritius
6.	Evaluation of the dengue-like syndrome clinical case definition, Reunion
7.	Emergency room consultations for epilepsy, stroke, asthma, and by tourists, Reunion
8.	Asthma burden in hospitals, Reunion
9.	Risk factors for multidrug-resistant staphylococcus aureus, Seychelles
10.	Risk factors for leptospirosis, Seychelles

**Outbreak investigations**: The fellows investigated 36 outbreaks (some were investigated by more than one FETP-OI fellow) either as principal or co-investigators. Of the investigated outbreak alerts 31% were related to foodborne epidemics, 28% to plague, 14% to malaria, 8% to “unknown illness”, and 19% to other illnesses (diarrhoea, dengue, conjunctivitis, haemorrhagic fever, skin rash and measles) Table 3.

**Epidemiological studies**: The first two FETP-OI cohort fellows worked on 18 different epidemiological studies Table 3. Most epidemiological studies were descriptive (45%) and treated diverse pathologies such as asthma, stroke, epilepsy, sport accidents, leptospirosis, gastroenteritis, and malaria. Four risk factor studies (22%) were planned and/or performed on plague, leptospirosis, tuberculosis and multidrug-resistant staphylococcus aureus. The remaining studies (33%) were of varied nature including evaluation of the clinical case definition of dengue-like syndrome, vaccination coverage evaluation regarding the extended programme of childhood immunisation, a literature review on antimicrobial resistance, and the impact of malaria control measures.

**Scientific communication**: FETP-OI fellows' activities have resulted in 26 oral and 15 poster presentations, including three oral and six poster presentations at the European Scientific Conference on Applied Infectious Disease Epidemiology (ESCAIDE 2013, 2015) and one oral presentation at the Global TEPHINET conference 2015. FETP-OI fellows have published four articles in regional Public Health newsletters (Bulletin de Veille Sanitaire, Cire océan Indien) [22, 23] and two are in process. There have been no publications yet in international peer-reviewed scientific journals (two are currently being submitted and more are planned).

**Transfer of competences**: In 24 instances FETP-OI fellows performed teaching activities, principally during FETP-OI training courses (14 or 59%). Of the remaining training activities, seven (29%) treated epidemiological surveillance and the remaining three (12%) other Public Health epidemiology subjects (literature review, outbreak investigation and the questionnaire in outbreak investigations).

**Fellows & career prospects**: The mean age of the 15 fellows that have finished the 24 months FETP-OI was 42 years (38 for the first and 45 for the second cohort) and seven (47%) were women. Eleven (73%) were medical doctors, and there were one environmental health officer, nurse, laboratory technician and Public Health officer respectively. Their positions after the programme are distributed as follows: 12 (80%) work in national or regional government positions, six of whom have had promotions after finishing the programme, one works at the Health Surveillance Unit of the Indian Ocean Commission, one at the Pasteur Institute in Madagascar, and one at the World Health Organization office in Madagascar. FETP-OI graduates have been involved in teaching for consecutive cohorts and supervising of FETP-OI projects. Several of them participate actively in the weekly regional network teleconference, which has further ensured the reinforcement of the network.

## Discussion

In its first four years, the FETP-OI has contributed to establishing a regional force of intervention consisting of field epidemiologists and trained supervisors using the same technical language and epidemiological methods. We have learned a number of lessons from the first two FETP-OI cohorts. Not all planned surveillance system set-up or research studies in the Comoros and Madagascar could be implemented due to lack of resources. Mauritius and Seychelles have had much fewer outbreak alerts allowing the fellows there to gain competencies in this area. It is possible to send fellows from these two countries on missions to Madagascar and Comoros, but so far, we haven't realised this. Due to political instability, the supervision of fellows within their host site was not always guaranteed. We believe it is crucial that host site supervisors devote at least 10% of their working time to close supervision of FETP-OI fellows to guide them in all methodological and operational aspects of their work. In some countries, notably Comoros and Madagascar, electricity, internet connections and telephone networks are unreliable. FETP-OI fellows receive financial support to try and ensure availability of communication means. The lack of academic accreditation of the FETP-OI diploma has been a deterrent for candidates from Mauritius and Seychelles. We have investigated possibilities of getting the programme recognised at university level, but this remains a challenge for the future. In the meantime, for the selection of the third cohort this lack of accreditation and main goal of the FETP-OI (gaining expertise in field epidemiology) was emphasized during the intake interviews.

**Limitations**: We believe the FETP-OI at present has three main limitations. So far, the great majority of FETP-OI projects consist of descriptive epidemiology. While this is the basis, and we consider it crucial that all graduates are capable of performing good descriptive analyses, there is a lack of analytical studies that we hope to address in the next years. Secondly, until today there have been no publications of FETP-OI work projects in scientific peer-reviewed international journals. Numerous papers are underway and we are confident they will be published. We need to share our results rapidly and render our work results more visible to the worldwide scientific community. The current funding of our programme comes to an end in 2017 and future funding is currently sought for. There is still a dire need of experienced and trained field epidemiologists to lead epidemiological surveillance, Public Health response and outbreak investigations in the Indian Ocean countries, and it is important to increase the number of individuals trained to respond to regional Public Health events. In the short term, our aim is to replace the current international experts in the Surveillance and Response unit of the IOC by FETP-OI graduates from the region.

## Conclusion

The FETP-OI has contributed to reinforcing surveillance and response capacities in the Indian Ocean. Numerous surveillance, outbreak investigation and research studies have been finalised and used for decision making in the Indian Ocean countries. Technically and financially sustainable FETP-OI work projects help addressing public health priorities. All fellows that have finished the FETP-OI have continued working in Public Health. The 3rd cohort consisting of nine public health professionals (78% women, mean age 36 years) started in November 2015. It includes five medical doctors, two veterinary clinicians, one nurse and one biology technician. More remains to be done within our aim to reinforce the One Health collaboration to ensure a comprehensive approach to epidemiological surveillance & preparedness, and securing the financial support to continue the field epidemiology training in the Indian Ocean region is a priority.

### What is known about this topic

- Regional or international collaboration is crucial to for health response preparedness;
- FETPs train competent field epidemiologists to strengthen the Public Health workforce as well as systems for disease surveillance, alert and response;
- An important additional asset of these programmes is the creation of solid collaboration and exchange networks.

### What this study adds

- The FETP-OI has created a regional force of intervention consisting of field epidemiologists and trained supervisors using the same technical language and epidemiological methods;
- A technically and financially sustainable FETP-OI project can help addressing public health priorities of the Indian Ocean region;
- The FETP-OI's numerous surveillance, outbreak investigation and research studies have been used for decision making in the Indian Ocean countries and reinforced surveillance and response capacities in the Indian Ocean.
